# Association of Baseline Cardiovascular Diseases with 5-Year Knee and Hip Osteoarthritis Progression in Non-Obese Patients: Data from the KHOALA Cohort

**DOI:** 10.3390/jcm10153353

**Published:** 2021-07-29

**Authors:** Camille Roubille, Joël Coste, Jérémie Sellam, Anne-Christine Rat, Francis Guillemin, Christian H. Roux

**Affiliations:** 1 Department of Internal Medicine, CHU Montpellier, Montpellier University, 34295 Montpellier, France; c-roubille@chu-montpellier.fr; 2 PhyMedExp, University of Montpellier, INSERM U1046, CNRS UMR 9214, CEDEX 5, 34295 Montpellier, France; 3 Biostatistics and Epidemiology Unit, Cochin Hospital, Paris University, 75014 Paris, France; joel.coste@aphp.fr; 4 Department of Rheumatology, Hôpital Saint-Antoine, AP-HP, Sorbonne University, 75006 Paris, France; jeremie.sellam@aphp.fr; 5 Inserm UMRS_938, Hôpital Saint-Antoine, 75012 Paris, France; 6 Department of Rheumatology, Caen University Hospital, Caen Normandie University, 14033 Caen, France; rat-ac@chu-caen.fr; 7 APEMAC, Université de Lorraine, 54000 Nancy, France; francis.guillemin@chru-nancy.fr; 8 CIC Clinical Epidemiology, CHRU Nancy, Inserm, Université de Lorraine, 54000 Nancy, France; 9 Department of Rheumatology, Nice Hospital, University Cote d’Azur, 06003 Nice, France; 10 Laboratory LAMHESS, EA6312. IBV CNRS IMR 7277 INSERM U1091 UNS, 06003 Nice, France

**Keywords:** osteoarthritis, radiological progression, comorbidities, cardiovascular diseases

## Abstract

We aimed to explore the relationship between comorbidities and the structural progression in symptomatic knee and/or hip osteoarthritis (OA) patients. We analyzed the 5-year outcome of non-obese participants (body mass index (BMI) < 30 kg/m^2^) from the KHOALA cohort having symptomatic hip and/or knee OA (Kellgren and Lawrence (KL) ≥ 2). The primary endpoint was radiological progression, defined as ΔKL ≥ 1 of the target joint at 5 years. The secondary outcome was the incidence of total knee or hip replacement over 5 years. Dichotomous logistic regression models assessed the relationship of comorbidities with KL progression and joint replacement while controlling for gender, age and BMI. Data from 384 non-obese participants were analyzed, 151 with hip OA and 254 with knee OA. At 5 years, cardiovascular diseases (CVD) were significantly associated with the 5-year KL change in both knee (OR = 2.56 (1.14–5.78), *p* = 0.02) and hip OA (OR = 3.45 (1.06–11.17), *p* = 0.04). No significant relationship was found between any type of comorbidities and knee or hip arthroplasty. This 5-year association between CVD and radiological progression of knee and hip OA in non-obese participants argue for an integrated management of CVD in knee and hip OA non-obese patients.

**Significance and innovations:**

1. What was already known before this study

Osteoarthritis (OA) subjects are more likely to develop some comorbidities.

Increasing evidence suggests that cardiovascular morbidity and mortality are higher in subjects with OA than without OA.

2. What was learned from this study

Cardiovascular diseases were associated with both knee and hip OA KL progression in non-obese participants. 

Such association was not found for arthroplasty implantation.

Cardiovascular diseases may have a specific impact on OA structural progression in non-obese patients, supporting their appropriate management.

## 1. Introduction

Osteoarthritis (OA) and cardiovascular diseases (CVD) are the most prevalent conditions in developed countries, contributing to the global health burden worldwide, due to pain, disability, loss of work and cost of treatment [1,2]. The prevalence of OA increases due to the aging population, as does the prevalence of many other disabling conditions co-occurring or co-existing with OA, named “comorbidities”, which may worsen patient global well-being.

OA subjects are more likely to develop some comorbidities [3,4]. Comorbidities impact morbidity, increase mortality, impair quality of life and also affect response to treatment, while increasing complexity of management of OA patients and its costs [5,6]. Comorbidity count has been associated with activity limitation, pain and poor health perception [7,8]. Patients with OA experience many comorbidities, especially metabolic and cardiovascular ones, which may have some specific impact on OA progression [9]. Around 40% of OA patients suffer from CVD [10], which have been associated with deteriorated physical functioning [11]. Increasing evidence suggests that cardiovascular morbidity and mortality are higher in OA populations than in subjects without OA [12], especially in knee and hip OA patients with severe disability [13]. In a recent meta-analysis, OA patients have been reported to have a higher cardiovascular (CV) risk as compared to controls [14], with an increased risk of myocardial infarction (RR = 1.22; 95% CI: 1.02–1.45) and of stroke (RR = 1.22; 95% CI: 1.02–1.45). Low-grade systemic inflammation may contribute to both OA and CVD [15].

One of the most challenging concerns in OA is the progression of structural damages. To date, OA treatment remains only symptomatic. Thus, identifying the factors contributing to disease progression may enable specific management and prevention. So far, known risk factors for progressive knee OA are local articular biomechanical factors, such as joint injury, or malalignment, and systemic factors including age, sex, genetics and obesity [16]. No conclusive evidence has been provided in favor to the impact of specific comorbidities such as CVD on structural damage in knee or hip OA. Moreover, given that both OA and CVD share common risk factors, especially obesity, it seems challenging to control for confounding effect of obesity by exploring the impact of comorbidities on OA progression in specifically non-obese population. Therefore, this longitudinal study aimed to explore the relationship between comorbidities and the progression of structural changes in symptomatic knee and/or hip OA non-obese patients over 5 years.

## 2. Patients and Methods

### 2.1. The KHOALA Cohort

The KHOALA (Knee and Hip OsteoArthritis Long-term Assessment) cohort is a French prospective multicenter observational cohort that included 878 participants, aged 40 to 75 years, with confirmed symptomatic hip and/or knee OA at baseline, fulfilling the American College of Arthritis criteria for knee and hip OA, and with Kellgren and Lawrence (KL) ≥ 2 on either knee or hip (for instance, a participant with knee KL ≥ 2 could be included while having a hip KL score < 2). The objective, design and characteristics of the cohort have been previously described [17]. Briefly, patients were recruited by an OA multiregional population-based survey conducted in France from 2007 to 2009. Some clinical, biological, functional and radiographic data were recorded. In particular, at baseline, medical history, physical examination (including weight and height), and data about comorbidities were collected, as well as biological parameters (including lipids levels) measured using standard methods. Participants underwent weight-bearing antero-posterior, posteroanterior semiflexed and axial views of both knees and/or antero-posterior pelvis and Lequesne views of both hips according to symptomatic joints. Target hip and knee tibio-femoral compartment were scored based on KL grades (from 0: no OA, to 4: severe). Radiographs were performed at baseline, 3 and 5 years, and were read centrally by the same readers blinded to clinical condition at each assessment time.

All included participants were evaluated annually, with either clinical investigation (performed at baseline, year 3, and then year 5), or self-reported questionnaires by mail. All participants gave their written informed consent to be included in the cohort before inclusion and the ethics committee CPP Est III gave approval for the cohort study (No. 07.01.01) registered at ClinicalTrials.gov (No. NCT00481338).

Patients were not involved in the design, or conduct, or reporting or dissemination plans of our study. Once the study has been published, participants will be informed of the results through a dedicated information letter.

### 2.2. Study Design and Outcomes

Participants with a 5-year follow-up were included in the present study. Participants could be analyzed either for knee OA or hip OA or both. Radiological severity of the target joint was assessed by the Kellgren and Lawrence score, ranging from 0 to 4 [18] (the maximum grade at either side was used). For those with a KL score = 4 at the time of inclusion, the contralateral joint was analyzed if it was scored KL ≥ 2. Furthermore, given the close relationship between obesity and the different comorbidities analyzed, especially metabolic and cardiovascular ones, obese participants with a BMI > 30 kg/m^2^ were excluded from the analysis.

Various comorbidities were collected at inclusion: CVD excluding hypertension (i.e., coronary artery disease, heart failure, stroke, lower limb arteriopathy), hypertension, diabetes, hypercholesterolemia, hypertriglyceridemia, osteoporosis, digestive (e.g., gastroesophageal reflux disease, ulcer), pulmonary (e.g., asthma, chronic obstructive pulmonary disease), and psychiatric (depression, anxiety) diseases. Comorbidities were declarative and defined as “presence” or “absence” at inclusion, as assessed by the self-reporting Groll functional comorbidity index [19], except for hypertension (presence when blood pressure measure > 140/90 mmHg) and checked according to drug prescriptions. The database for the present study was locked in 2015 at the 5-year time point.

The primary endpoint was structural radiological progression, which was defined by the increase of at least one point of KL (ΔKL ≥ 1) at 5 years. The secondary outcome was the incidence of total knee or hip replacement over 5 years. Structural progression (KL progression) and OA severity (cumulative incidence of joint replacement over 5 years) were explored for knee OA and hip OA.

### 2.3. Statistical Analysis

Descriptive statistics were used to characterize baseline data of the study population, which are presented as mean ± standard deviation (SD) or percentage (%) where appropriate.

Dichotomous logistic regression models were used to examine the relationship of comorbidities with OA at baseline and with outcomes (KL progression, joint replacement) while controlling for gender, age and BMI. Therefore, we tested each comorbidity against no comorbidity with adjustment for age, gender and BMI as confounding variables. Analyses were performed separately in hip and knee OA, using SAS 9.4 software (SAS Institute Inc., Cary, NC, USA).

## 3. Results

### 3.1. Study Population

**Figure 1:**
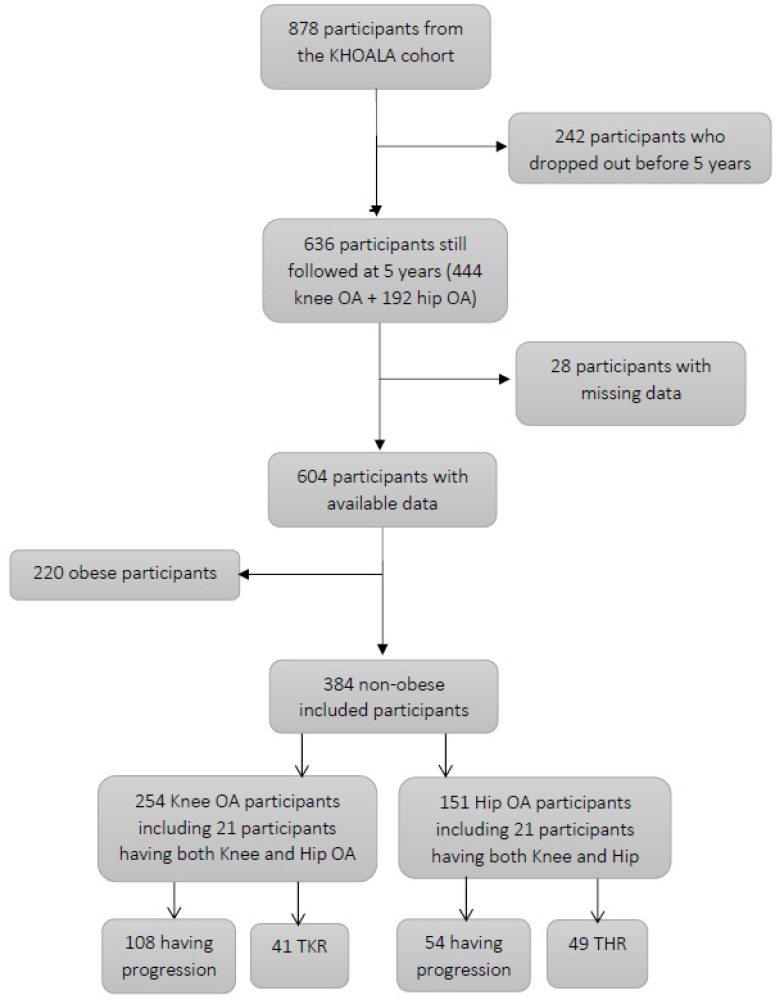
Study design.

Six hundred and thirty-six participants were still followed at 5 years, including 444 knee OA patients and 192 hip OA patients. Among them, 384 non-obese (BMI < 30 kg/m^2^) participants were included in the present study (Figure 1). The study population comprised 254 participants with knee OA and 151 with hip OA, including 244 women, mean age of 60.6 ± 8.8 years. In this total population, 197 (51.3%) reported no comorbidity, 121 (31.5%) had one comorbidity, 53 (13.8%) had two comorbidities, 13 (3.5%) had three or four comorbidities. One hundred and twenty-two knee OA patients had at least one comorbidity (48%), similarly to 72 hip OA patients (47.7%). First-visit characteristics of the study population are presented in Table 1. We found no association between comorbidity and baseline knee or hip OA (Table 2).

### 3.2. Association of Baseline Comorbidities with Structural Progression

One hundred and eight knee OA participants over 254 experienced some structural progression (ΔKL ≥ 1) at 5 years (42.5%) and 41 had knee arthroplasty. Fifty-four hip OA participants over 151 experienced some structural progression (ΔKL ≥ 1) at 5 years (35.3%) and 49 had hip arthroplasty.

CVD were significantly associated with the 5-year KL change in both knee OA (OR = 2.56 (1.14–5.78), *p* = 0.02) and hip OA (OR = 3.45 (1.06–11.17), *p* = 0.04) (Table 3). Hypertriglyceridemia was associated with radiographic progression of hip OA (*p* = 0.02) while it was inversely associated with radiographic progression of knee OA (*p* = 0.05). Moreover, pulmonary comorbidities were inversely associated with radiographic progression of hip OA (*p* = 0.05). No other comorbidities showed a significant association on knee or hip OA structural progression. No significant relationship was found between any type of comorbidities and knee or hip arthroplasty. Moreover, no significant association was found between having more than three comorbidities compared to having three or less, and KL change or arthroplasty.

## 4. Discussion

The aim of the present study was to evaluate the impact of various comorbidities on knee and hip structural changes and on the need for joint replacement in non-obese participants from a cohort of symptomatic knee and/or hip OA. In this cohort based on a real-life setting monitored for 5 years, we found a significant association between CVD and both knee and hip OA structural progression in non-obese participants, as evaluated by KL progression. Such association was not found for the incidence of arthroplasty. Although still speculative at this time, these findings suggest a specific impact of CVD on OA structural progression in non-obese patients, providing important insight that may help in understanding the patient population that might be more at risk to progress and perhaps may benefit from an integrated management. Given that obesity has largely been associated with OA progression, investigating the relationship between comorbidities and structural changes or joint replacement in non-obese patients remains under-studied and appears to be highly relevant.

There are several hypotheses for the potential impact of CVD on OA progression, independently of BMI and obesity, as suggested by our present results. The potential interaction of those two conditions is probably multifactorial. OA and CVD obviously share common risk factors and pathogenic mechanisms, such as inflammation, metabolic factors, and limitation of physical activity. Indeed, low-grade systemic inflammation or meta-inflammation may be the common cornerstone pathway leading to OA, atherosclerosis and CVD, as suggested by the association between hand OA and CVD [20]. OA radiographic severity has also been associated with C-reactive protein (CRP) serum levels [21], as well as OA pain severity [22]. Some inflammatory factors may contribute to both OA progression and vascular dysfunction, making meta-inflammation the pivotal link interconnecting OA progression and cardiovascular comorbidities. However, such an association could be multifactorial and not solely related to metabolic factors, given our results in non-obese participants. In addition, while OA affects the ability to exercise, a sedentary lifestyle is a well-known cardiovascular risk factor. OA may also reduce the potential for rehabilitation after cardiovascular events. On the other hand, suffering from certain CVD such as heart failure or stroke with muscle weakness and sarcopenia, also contributes to reduced physical activity, and may increase the risk of OA progression, leading to a vicious circle. Importantly, OA structural changes comprise not only cartilage loss but also loss of the integrity of surrounding tissues, such as subchondral bone remodeling. Therefore, peripheral low blood flow related to endothelial dysfunction may play an important role in OA progression, with subchondral ischemia leading to impaired cartilage nutrition, osteocyte apoptosis, bone resorption and articular damage [23,24]. All these mechanisms may underpin the relationship between CVD and OA.

Nevertheless, on the contrary to rheumatoid arthritis where CV risk is well-established and subsequent management fully recommended, the increased CV risk in OA patients seems under-recognized by patients and health care providers. Our data suggest that CVD may have an impact on knee OA progression. However, literature about the link between CVD per se and OA in terms of radiographic progression remains scarce [20].

As regards cardiovascular risk factors, participants with OA are prone to develop hypertension, which is a well-known risk factor for CVD. Hypertension has been reported as the most frequent comorbidity in OA patients. In the ROAD study, Yoshimura et al. showed that hypertension was a strong risk factor for OA progression (RR = 1.47) [9]. In a recent meta-analysis, hypertension increased by 1.5 and 2.0 the risk of radiographic and symptomatic knee OA respectively [25]. In animal models, hypertensive rats spontaneously developed more severe subchondral bone damage compared to normotensive rats, independently of obesity [26]. However, the relationship between hypertension and OA progression in non-obese patients remains unclear, and here we found only a trend toward an association between hypertension and radiographic progression in knee OA. Therefore, further studies are needed.

Diabetes has been associated to OA [27], with a possible role of advanced glycation end products (AGEs), oxidative stress and meta-inflammation as pathological pathways. Although some studies have reported that type 2 diabetes may be associated to knee OA radiographic progression [28] and arthroplasty [29], the role of diabetes in OA progression remains controversial. However, a recent review showed that this relationship is not independent from obesity [30]. Here, we did not find any association between diabetes and progression in non-obese patients.

With regard to hypertriglyceridemia, we found a contrasting association with the progression of OA of the knee or hip. In MRI studies, triglycerides level has been associated with the incidence of bone marrow lesions in knee OA, but not with cartilage volume loss [31]. Further studies are needed to explore this point, particularly in non-obese participants.

As regards arthroplasty, finding no association between any comorbidity and knee or hip replacement is not uncommon. Previous studies reported that CVD may restrain surgeon’s decision in proceeding arthroplasty [32]. This point may explain why we found an association between CVD and OA progression while not finding such association with arthroplasty.

While our data point to more progression in knee OA patients suffering of CVD, it should be treated with caution and need to be confirmed in further studies. Limitations of the present study are those inherent to observational cohort studies, with potential confounding factors which could not be accounted for. Moreover, most data regarding comorbidities were declarative, with potential recall bias. The lack of information about potential CV treatments for CVD during follow-up may be another limitation. Finally, the moderate number of arthroplasties and the attrition of the cohort at 5 years can be considered as a limitation.

Our study has also a number of strengths. The KHOALA cohort is a representative sample of population-based participants with symptomatic knee and hip OA, including a large panel of participants. It allows exploration of the time-dependent progression of OA and need for arthroplasty, in a real-world setting, providing long follow-up time with a low rate of missing data or drop-out. Additionally, all radiographs were analyzed in a unique centralized radiology reading center. Finally, to reduce the confounding bias related to obesity, which is well-known to be closely associated with both OA progression and CVD, we chose to consider only non-obese participants, and data were adjusted on BMI. Indeed, in the context of personalized management of OA, it seems clinically and epidemiologically relevant to identify subsets of OA patients who need to be tightly monitored, and who might best benefit from a multidisciplinary approach. In non-obese knee OA patients, our data argue for a holistic CVD management including prevention, for instance with physical activity, and tight control of cardiovascular risk factors, that may be beneficial not only for cardiovascular reasons but also perhaps to delay OA progression.

Considering comorbidities in the management of OA patients may seem difficult to integrate into a classical visit focused on OA itself, especially because it would be time-consuming. Perhaps, priority should be given to the management of the comorbidities that require the most active care in OA patients, which could be CVD as suggested by our findings.

In summary, this 5-year data analysis of the KHOALA cohort revealed a significant association between CVD and structural progression in both knee and hip OA non-obese participants. These results support the integrated management of CVD in knee and hip OA patients.

## Figures and Tables

**Table 1:** Baseline participants demographic and clinical characteristics (data are presented as mean ± standard deviation (SD) or percentage (%) where appropriate).

	Total (BMI < 30) *n* = 384	Baseline Hip OA (BMI < 30) *n* = 151		Baseline Knee OA (BMI < 30) *n* = 254	
Explanatory Variables	*n* (%) or Mean ± SD	BMI < 25 kg/m^2^	25 < BMI < 30 kg/m^2^	BMI < 25 kg/m^2^	25 < BMI < 30 kg/m^2^
		*n* = 70	*n* = 81	*n* = 82	*n* = 172
Age (years)	60.6 ± 8.8	59.2 ± 8.4	61.6 ± 8.7	61.0 ± 8.8	61.6 ± 8.7
Gender (female)	244 (63.5)	47 (67.1)	50 (61.7)	64 (78%)	95 (21.4)
BMI	25.7 (2.7)	22.9 (1.8)	27.5 (1.5)	22.7 (1.4)	27.5 (1.4)
Smoking	70 (18.3)	16 (22.9)	11 (13.6)	12 (14.8)	34 (19.9)
Kellgren and Lawrence score *					
1		0 (0)	0 (0)	6 (7)	6 (3)
2		47 (67)	53 (65)	42 (51)	78 (45)
3		19 (27)	24 (30)	21 (26)	46 (27)
4		4 (6)	4 (5)	13 (16)	42 (25)
Diabetes	18 (4.7)	2 (2.9)	3 (1.6)	2 (2.4)	11 (6.4)
Cardiovascular diseases	46 (12.0)	2 (2.9)	14 (17.3)	11 (13.4)	21 (12.1)
Hypertension	128 (33.3)	18 (25.7)	26 (32.1)	21 (25.6)	69 (40.1)
Hypercholesterolemia	32 (8.4)	5 (7.4)	8 (9.9)	3 (3.7)	17 (9.9)
Hypertriglyceridemia	113 (29.7)	9 (13.2)	30 (37)	10 (12.2)	67 (39.2)
Osteoporosis	24 (6.3)	6 (8.6)	3 (3.7)	7(8.5)	10 (5.8)
Pulmonary comorbidities	30 (7.8)	6 (8.6)	5 (6.2)	6 (7.3)	14 (8.1)
Gastrointestinal comorbidities	73 (19.0)	14 (20)	18 (22)	11 (13.4)	32 (18.6)
Psychiatric comorbidities	72 (18.8)	14 (20)	13 (16.1)	14 (17.0)	32 (18.6)
Nb. of comorbidities					
0	197 (51)	40 (57)	39 (48)	47 (57)	85 (49)
1	121 (32)	18 (26)	28 (35)	23 (28)	58 (34)
2	53 (14)	9 (13)	13 (16)	9 (11)	23 (13)
≥3	13 (3)	3 (4)	1 (1)	3 (4)	6 (3)

* Maximal Kellgren and Lawrence score.

**Table 2:** Relationship of comorbidities with hip or knee OA at baseline; absence of comorbidity is the reference category. Odds ratio adjusted for age, gender and BMI.

	Total *n* = 384	Baseline Hip OA (BMI < 30) *n* = 151			Baseline Knee OA (BMI < 30) *n* = 254		
Comorbidity	*n* (%)	Adjusted OR	95% CI	*p*-Value	Adjusted OR	95% CI	*p*-Value
Diabetes	18 (4.7)	0.63	0.22–1.84	0.40	1.14	0.39–3.32	0.81
Cardiovascular diseases	46 (12.0)	0.85	0.44–1.64	0.63	1.05	0.53–2.09	0.88
Hypertension	128 (33.3)	0.72	0.45–1.17	0.19	1.22	0.74–2.01	0.43
Hypercholesterolemia	32 (8.4)	1.17	0.55–2.48	0.68	0.73	0.34–1.57	0.42
Hypertriglyceridemia	113 (29.7)	0.82	0.51–1.33	0.43	0.94	0.57–1.55	0.81
Osteoporosis	24 (6.3)	0.86	0.35–2.08	0.74	1.33	0.52–3.41	0.55
Pulmonary comorbidities	30 (7.8)	0.92	0.42–2.00	0.83	0.97	0.44–2.16	0.94
Gastrointestinal comorbidities	73 (19.0)	1.30	0.77–2.20	0.32	0.63	0.37–1.08	0.10
Psychiatric comorbidities	72 (18.8)	0.95	0.55–1.62	0.85	0.83	0.48–1.43	0.50
Nb. of comorbidities > 3	13 (3.4)	0.68	0.20–2.31	0.54	1.08	0.32–3.66	0.90

Abbreviations: KHOALA: Knee and Hip OsteoArthritis Long-term Assessment cohort; OA, osteoarthritis; THR: total hip replacement; TKR: total knee replacement.

**Table 3:** Risk of progression and arthroplasty of knee and hip osteoarthritis during the 5-year follow-up associated with studied comorbidities (absence of comorbidity is the reference category). * Odds ratios and 95%confidence intervals adjusted for age, gender and BMI.

Knee OA									
	Radiographic Progression ^a^			Arthroplasty ^b^			Radiographic Progression or Arthroplasty ^e^		
Comorbidity	OR *	95% CI	*p*-Value	OR *	95% CI	*p*-Value	OR *	95% CI	*p*-Value
Diabetes	1.17	0.37–3.68	0.79	1.23	0.32–4.74	0.76	0.92	0.34–2.89	0.87
Cardiovascular diseases	2.56	1.14–5.78	0.02	1.19	0.47–3.01	0.71	2.85	1.26–6.44	0.01
Hypertension	1.59	0.89–2.88	0.12	1.28	0.62–2.64	0.50	1.46	0.84–2.55	0.19
Hypercholesterolemia	1.80	0.72–4.52	0.21	1.41	0.52–3.83	0.50	1.30	0.53–3.28	0.57
Hypertriglyceridemia	0.55	0.30–1.00	0.05	1.44	0.71–2.92	0.32	0.72	0.41–1.25	0.25
Osteoporosis	0.54	0.18–1.63	0.27	0.90	0.24–3.41	0.88	0.53	0.19–1.48	0.23
Pulmonary comorbidities	2.08	0.81–5.34	0.13	0.83	0.23–3.00	0.78	2.24	0.83–6.07	0.11
Gastrointestinal comorbidities	1.18	0.63–2.23	0.60	0.80	0.34–1.88	0.61	1.17	0.63–2.19	0.61
Psychiatric comorbidities	0.86	0.44–1.69	0.66	1.31	0.60–2.85	0.50	0.83	0.44–1.56	0.57
Nb. Of Comorbidities > 3	1.51	0.42–5.49	0.53	1.04	0.21–5.24	0.96	1.30	0.35–4.86	0.69
**Hip OA**									
	**Radiographic Progression ^c^**			**Arthroplasty ^b^**			**Radiographic Progression or Arthroplasty ^f^**		
**Comorbidity**	OR *	**95% CI**	***p*-Value**	OR *	**95% CI**	***p*-Value**	OR *	**95% CI**	***p*-Value**
Diabetes	0.31	0.03–2.77	0.29	0.39	0.05–3.10	0.38	0.34	0.26–1.86	0.20
Cardiovascular diseases	3.45	1.06–11.17	0.04	0.61	0.20–1.81	0.37	2.37	0.77–7.37	0.13
Hypertension	0.98	0.45–2.12	0.96	0.73	0.36–1.48	0.38	0.99	0.49–2.01	0.98
Hypercholesterolemia	0.65	0.19–2.22	0.49	0.41	0.09–1.81	0.24	0.42	0.14–1.25	0.12
Hypertriglyceridemia	2.51	1.15–5.48	0.02	1.01	0.49–2.07	0.99	2.10	0.99–4.42	0.05
Osteoporosis	0.39	0.08–1.95	0.25	0.66	0.18–2.38	0.52	0.46	0.14–1.55	0.21
Pulmonary comorbidities	0.12	0.02–0.97	0.05	0.76	0.22–2.63	0.66	0.21	0.05–0.79	0.02
Gastrointestinal comorbidities	0.77	0.32–1.83	0.55	1.35	0.65–2.78	0.42	0.68	0.32–1.43	0.32
Psychiatric comorbidities	0.94	0.38–2.31	0.90	0.88	0.40–1.94	0.76	0.96	0.32–2.13	0.29
Nb. Of Comorbidities > 3	<0.01	<0.01–>999	0.98	0.44	0.05–3.54	0.44	0.19	0.02–1.89	0.69

^a^ n = 258; ^b^ n = 384; ^c^ n = 153; ^e^ = 274; ^f^ = 175.

## Data Availability

Data could be available on reasonable request.
